# Modeling Analysis of a Polygeneration Plant Using a CeO_2_/Ce_2_O_3_ Chemical Looping

**DOI:** 10.3390/ma16010315

**Published:** 2022-12-29

**Authors:** Greta Magnolia, Massimo Santarelli, Domenico Ferrero, Davide Papurello

**Affiliations:** 1 Department of Energy (DENERG), Politecnico di Torino, Corso Duca Degli Abruzzi, 24, 10129 Turin, Italy; 2 Energy Center, Politecnico di Torino, Via Borsellino 38/18, 10129 Turin, Italy

**Keywords:** chemical looping, polygenerative system, ceria oxides, biological methane, SOFC, CS, DME

## Abstract

In the current context of complexity between climate change, environmental sustainability, resource scarcity, and geopolitical aspects of energy resources, a polygenerative system with a circular approach is considered to generate energy (thermal, electrical, and fuel), contributing to the control of CO_2_ emissions. A plant for the multiple productions of electrical energy, thermal heat, DME, syngas, and methanol is discussed and analyzed, integrating a chemical cycle for CO_2_/H_2_O splitting driven using concentrated solar energy and biomethane. Two-stage chemical looping is the central part of the plant, operating with the CeO_2_/Ce_2_O_3_ redox couple and operating at 1.2 bar and 900 °C. The system is coupled to biomethane reforming. The chemical loop generates fuel for the plant’s secondary units: a DME synthesis and distillation unit and a solid oxide fuel cell (SOFC). The DME synthesis and distillation unit are integrated with a biomethane reforming reactor powered by concentrated solar energy to produce syngas at 800 °C. The technical feasibility in terms of performance is presented in this paper, both with and without solar irradiation, with the following results, respectively: overall efficiencies of 62.56% and 59.08%, electricity production of 6.17 MWe and 28.96 MWe, and heat production of 111.97 MWt and 35.82 MWt. The fuel production, which occurs only at high irradiance, is 0.71 kg/s methanol, 6.18 kg/s DME, and 19.68 kg/s for the syngas. The increase in plant productivity is studied by decoupling the operation of the chemical looping with a biomethane reformer from intermittent solar energy using the heat from the SOFC unit.

## 1. Introduction

**Figure 1:**
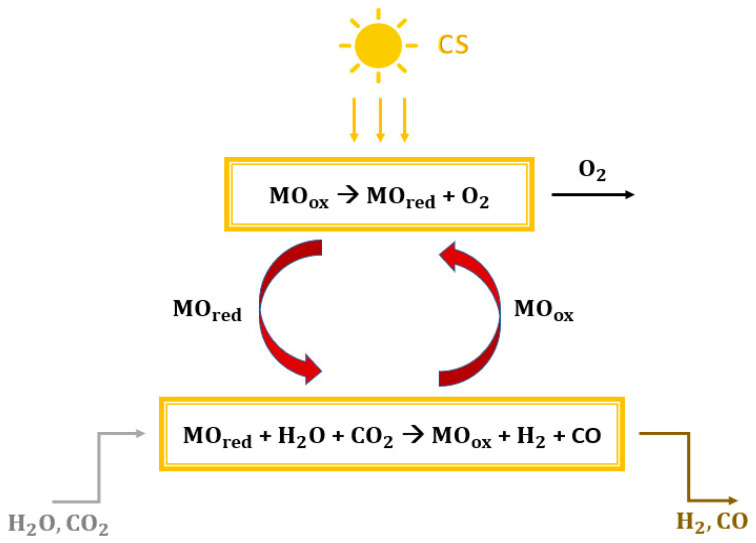
Generic scheme of a thermochemical splitting cycle fed by concentrated solar energy.

Greenhouse gas emissions, climate change, and carbon scarcity are serious problems that require a strong focus. The sixth assessment report by the IPCC has been published, in which the role of anthropogenic activities is updated [1,2]. To reduce this impact on the environment, it was decided to limit the increase in global average temperature to 1.5 °C above pre-industrial levels [3]. The power generation sector plays a key role in reducing greenhouse gas emissions [4,5]. The use of solar energy in power generation could be a key tool to limit the use of fossil fuels. Solar radiation is highly available, globally distributed, clean, and renewable. However, the intermittent nature and recurring mismatch between consumers and direct supply are the main factors limiting its expansion. These problems can be overcome by non-fossil solar fuels [6,7], which store solar energy [8]. In this paper, solar energy is stored using a mixture of carbon monoxide and hydrogen. This gas mixture can be used as a direct fuel in a gas turbine [9,10] and an internal combustion engine [11,12] or in a fuel cell to generate electricity [12]. Furthermore, the conversion of syngas into fuels and other chemicals makes it possible to produce carbon-neutral materials [13,14,15]. The production of dimethyl ether from syngas is becoming an increasingly attractive route. The scientific community regards DME as the fuel that could eliminate the dependence on fossil fuels [16]. This is due to its physical properties, which are very similar to those of liquefied petroleum gas (LPG, e.g., propane and butane), with a low environmental and health impact [17]. Moreover, DME distillation also produces methanol (CH_3_OH) after dimethyl ether synthesis from syngas [18]. The main methods for producing solar syngas are photobiological or photochemical conversion, thermochemical conversion, and electrochemical conversion. The first route occurs at relatively low temperatures by directly using solar energy from photons to power the process [19,20]. Electrochemical conversion processes consist of the co-electrolysis of CO_2_ and H_2_O in an electrolyzer that supplies electricity from photovoltaic systems or solar thermal power plants [21,22]. Thermochemical conversion processes use concentrating solar collectors (CSP) to concentrate solar radiation to a point where a receiver reactor is located [23,24]. The simplest thermochemical pathway for syngas production would be the direct one-step solar thermal dissociation of water and carbon dioxide (thermolysis). However, this route is not practical, because an extremely high temperature of more than 2200 °C is required and cannot be achieved, due to the limitations imposed by the reactor materials, the thermal losses, and the possible products recombination to avoid explosive mixtures [25,26,27,28]. One way to lower the reduction temperature is to use redox cycles [20,29]. Two phases characterize the basic and simplest thermochemical cycles [30,31,32], with overall efficiencies of around 40–50% [33,34]. Considering a metal oxide (MO) with different oxidation states, the two steps are shown in Figure 1:

1. Reduction reaction (1): the concentrated solar energy provides thermal heat. The metal oxide MOox undergoes thermal reduction and releases oxygen (MOred is obtained).
2. Oxidation reaction (2): the reduced metal oxide MOred reacts with water and carbon dioxide to achieve both the reoxidation of metal and the reduction of H_2_O and CO_2_ with the subsequent production of syngas.

MO_ox_ → MO_red_ + O_2_ (1)

MO_red_ + H_2_O + CO_2_ → MO_ox_ + H_2_ + CO (2)

**Figure 2:**
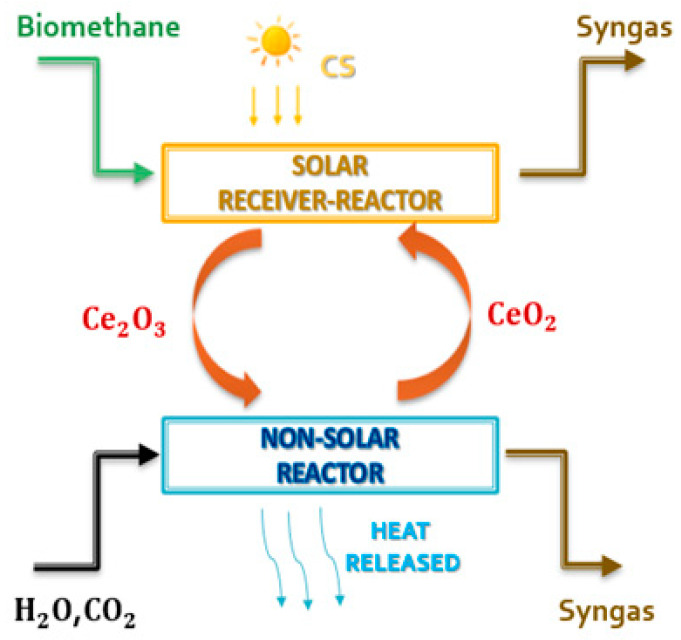
Schematic representation of the ceria chemical looping coupled with biomethane reforming.

Various metal-redox pairs have been investigated for the TSC processes. Ceria is currently considered the most attractive metal-redox pair for various reasons [35,36,37,38,39]: high crystallographic stability during extended thermal cycling (non-volatile cycle), high oxygen release and storage capacities (>100 mmol min^−1^ g^−1^), fast oxygen exchange rates, reversible shift between Ce^4+^ and Ce^3+^ oxidation states, and fast kinetics during thermochemical cycles as compared to other non-volatile metal oxides. However, the reduction reaction of pure ceria occurs only at very high temperatures (above 1400 °C) and very low pressures (between 100 and 200 mbar) [38]. The introduction of biomethane as a reducing agent (see Figure 2) could solve these problems [40,41,42,43] while preserving sustainability criteria, allowing the realization of an isothermal and isobaric cycle according to reactions (1) and (2).

(Reduction reaction)   2CeO_2_ + CH_4_ → CO + 2H_2_ + Ce_2_O_3_ (3)

(Oxidation reaction)   2Ce_2_O_3_ + CO_2_ + H_2_O → 4CeO_2_ + H_2_ + CO (4)

Other thermochemical processes to produce syngas could be [25]: steam or dry natural gas/biomethane reforming [44], and biomass or coal gasification [45]. Biomethane could be produced by different separation mechanisms for upgrading the biogas extracted from the organic fraction of MSW (e.g., pressure swing adsorption, cryogenic separation, and absorption techniques [46]).

Polygeneration systems could represent an efficient and cost-effective solution for the integration of solar thermochemical conversion processes [47]. These systems have gained increasing attention in recent years due to their high overall efficiency and the possibility to use waste heat streams to generate useful heat or to obtain additional useful power [33,48]. As a result, these solutions generate multiple products such as electricity, cooling, heat, freshwater, and chemicals from one or more input streams in a single system. Several examples of polygeneration systems integrated with solar thermochemical conversion units can be found in the literature. Kaniyal et al. [49] performed a polygeneration system analysis based on hybrid solar gasification of coal at atmospheric pressure to produce liquid fuels and electricity. To solve the problem of intermittent solar energy, they proposed the pressurized storage of upgraded syngas and oxygen that can be used during periods of low solar radiation. Liu et al. [50] conducted a thermodynamic study on a lignite polygeneration system powered by solar energy. They demonstrated that the use of solar energy in lignite drying pyrolysis and gasification can save 63.84% of lignite energy compared to conventional methods. Kong et al. studied a polygeneration plant based on an isothermal thermochemical cycle fed with concentrated solar energy and methane to produce electricity and methanol simultaneously. They found that the fossil fuel consumption to produce one unit mass of methanol was about 22 GJ/ton with an optimum efficiency of more than 44%. Bai et al. [51] carried out a techno-economic analysis of a polygeneration system based on solar thermal gasification of biomass to produce methanol and electricity. They concluded that the energy and exergy efficiencies could reach values of 56.09% and 54.86%, respectively.

All literature studies are characterized by the production of CO_2_ streams, making the system not exactly carbon neutral. These gas streams could be emitted into the atmosphere without proper and accurate carbon capture and sequestration processes.

As far as we know, there are no studies on zero-emissions polygeneration plants at an industrial scale. In this paper, a polygeneration system with the solar-driven thermochemical cycle is simulated, analyzed, and discussed.

## 2. Case Study: Polygeneration Plant

The plant is built with four units:

1. Chemical looping unit. This section is the driving force of the plant and produces the fuel for the other units. The CeO_2_/Ce_2_O_3_ redox couple associated with biomethane reforming is chosen (Figure 2). The cycle is unpressurized and isothermal at 900 °C and 1.2 bar. The reduction reaction takes place within a solar receiver via a solar tower, while the oxidation reaction takes place in a non-solar reactor. These two reactors operate simultaneously when solar energy is available. Under steady-state conditions, the reduction reactor is fed with 0.59 kmol/s of ceria with a particle size of 0.4–1 mm;
2. Fixed SOFC unit. The secondary unit is fed by the syngas produced in the CL reduction reactor. Electricity is generated in this unit;
3. DME synthesis and distillation unit integrated with biomethane reforming reactor fed by concentrated solar energy for CCU unit [52]. This is another secondary unit fed with syngas. The syngas is produced in the oxidation reactor of CL. DME, methanol, and syngas are produced from this unit;
4. Steam generation plant. There are five steam generators with heat recovery in the plant that use the hot flows of the system to generate steam. This steam is used to heat other cold process flows and to generate thermal energy to supply other consumers (e.g., a district heating network).

The chemical cycle can operate properly at sufficient irradiance to heat the reduction reactor at 900 °C, while at lower irradiance levels, the chemical cycle turns off. A comparison is made with a similar non-solar polygeneration system published in the literature [53]. An attempt to increase the system’s productivity by decoupling its operation from solar energy fluctuations is made. The results of the chemical cycle integrated into the system are compared with experimental [54] and modeling studies [41,55].

The proposed plant produces thermal energy, electricity, syngas, methanol, and dimethyl ether (DME), in two operating conditions:

(1) Operation on a day with a clear sky and irradiance sufficient for the CL operation;
(2) Operation on a day with low solar irradiance.

**Figure 3:**
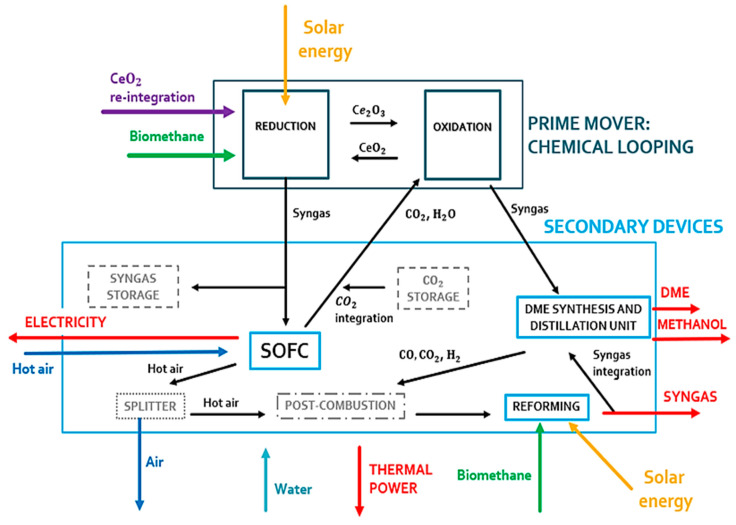
Prime mover and secondary devices of the polygeneration plant during a high-irradiance clear sky day.

In the first operating condition (see Figure 3), all components of the plant can work properly. The reduction reactor of the CL is oversized compared to the SOFC. An excess of syngas is obtained from the reduction reactor. This gas mixture is stored and used during periods of low solar irradiation. This storage system is fundamental to couple the solar syngas with the correct operation of the SOFC system. This system is very sensitive to load variations and transients due to its high operating temperature.

**Figure 4:**
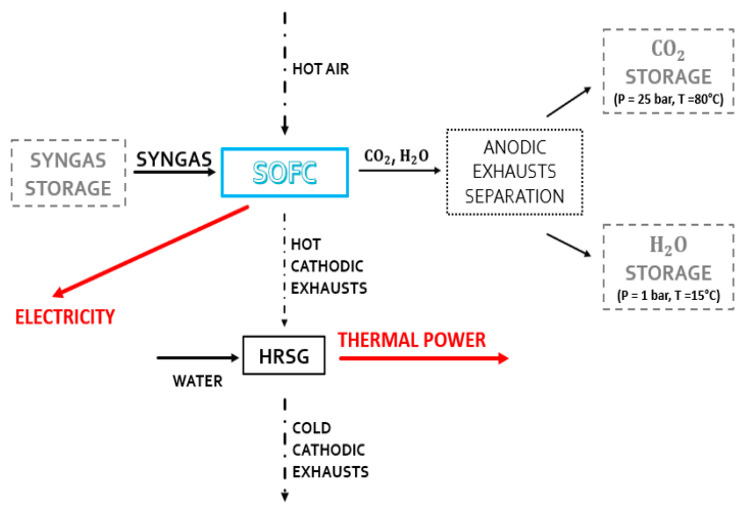
Active components when the system does not receive adequate solar radiation.

The uniqueness of the SOFC system’s operation is provided in the second operating condition plant. This system is fed by properly stored syngas (see Figure 4). When solar energy is not present, the chemical cycle and consequently the synthesis of DME, the distillation unit, and the reforming reactor do not come into operation. Therefore, the anode fluxes of the SOFC system are stored for reuse when the CL system is operational.

**Figure 5:**
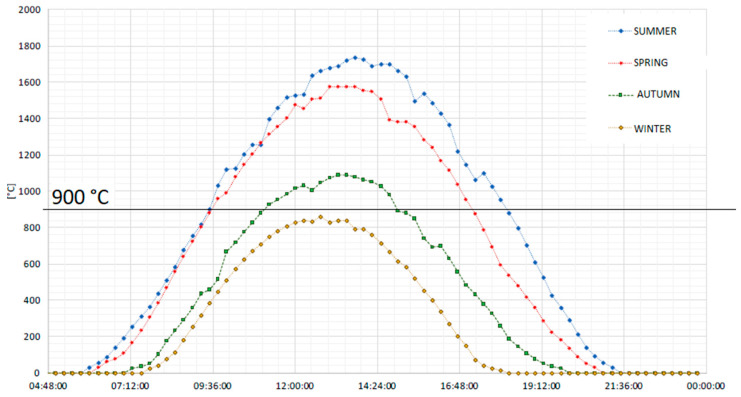
Average temperature profile along the year in the solar concentrated dish placed in Turin [57].

These different operating conditions are defined by considering the seasonal average daily temperatures of the reduction reactor. The experimental data for the modeling analysis are taken from the dish facility placed at the Turin-Energy Center [56]. Looking at the seasonal daily average temperature of the dish system, it is found that the temperature is higher/equal to 900 °C (see Figure 5):

(1) For 8 h and 30 min every day in the summer season;
(2) For 7 h and 40 min every day in the spring season;
(3) For 3 h and 40 min every day in the autumn season;
(4) Never active in winter.

In summary, the chemical cycle can operate around 21% of the year. The SOFC system is designed to operate continuously.

### 2.1. Modeling and Plant Simulation in Aspen Plus

In this study, the simulation tool used is Aspen Plus^®^ from AspenTech (v10, Bedford, MA, USA). In both plant schemes (Figure 3 and Figure 4), the plant is modeled mainly with built-in components and assumes chemical equilibrium in each component. The DME reactor is characterized by a kinetic approach. The main plant components are:

- Turbines, valves, coolers, distributors, mixers, heaters, compressors, heat recovery steam generators, flash units, and cyclones;
- RGIBBS reactor units are used to model the chemical looping oxidation and reduction reactors, the post-combustion unit, the SOFC anode, and the biomethane reforming reactor;
- Separator (coupled with a heat exchanger) for the cathode of the SOFC;
- RADFRAC columns used to model the DME distillation unit;
- RPLUG reactor coupled to the kinetic model Langmuir–Hinshelwood–Hougen–Watson (LHHW) is used to simulate the DME synthesis reactor with a catalytic behavior. In this unit, it is used for the thermodynamic properties of the Soave–Redlich–Kwong (SRK) equation of state (EOS). A similar approach was followed by Graaf et al. [58], where the SRK equation of state was used to model the chemical equilibrium of the methanol and water gas shift (WGS) reaction. This model is applied to binary components [59].

In the model, the flows used are conventional (H_2_O, CO_2_, H_2_, CO, CH_4_, N_2_, O_2_, CH_3_OH, and CH_3_OCH_3_) and fixed flows (oxygen carriers of chemical looping, CeO_2_ and Ce_2_O_3_). The Barin equation is used for the evaluation of the fixed flow [60]. For conventional flows, the Peng–Robinson–Boston–Mathias (PR-BM) properties method is used. This approach is widely used in the literature for the treatment of hydrocarbons, such as refining in petrochemical processes [61,62]. Table 1 lists the main assumptions used.

### 2.2. Plant Operating Components in a High-Irradiance Clear Sky Day

#### 2.2.1. Chemical Looping and Storage System

**Figure 6:**
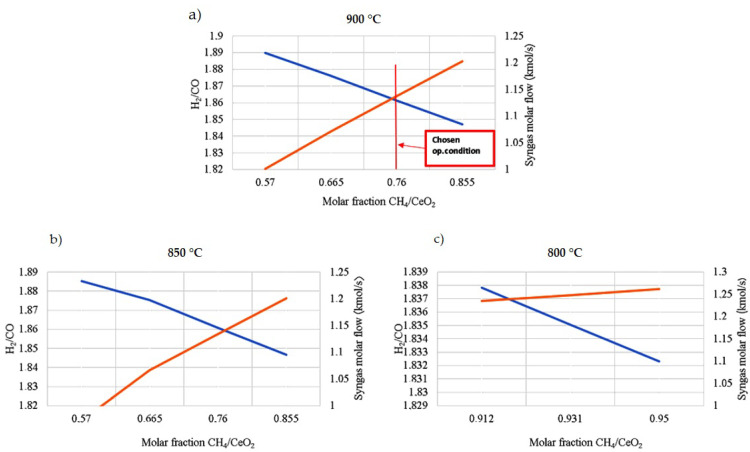
Operating conditions of the reduction reactor, syngas production (orange line), and H_2_/CO molar ratio (blue line) varying the operating temperatures and the CH_4_/CeO_2_ molar ratio. (**a**) The chosen operating conditions are 900 °C and a CH_4_/CeO_2_ molar ratio equal to 0.76 to satisfy all three requirements of this reactor; (**b**) at 850 °C; (**c**) at 800 °C. CeO_2_ reduction is complete only with CH_4_/CeO_2_ molar ratios higher than 0.912, with a much higher expenditure of biomethane if compared with the other two cases.

The constant-temperature and -pressure operating conditions for the CL process increase the efficiency by reducing losses associated with heat flows and high system pressures. Concerning the reduction reaction, the operating temperature and the amount of methane to be fed are chosen to consider the following three requirements (see Figure 6):

- The temperature should be compatible with a solar system;
- The complete reduction of the oxygen carrier to Ce_2_O_3_ must take place with a limited amount of biomethane;
- The desired syngas concentration has a molar ratio of H_2_/CO around 2. This molar ratio is more suitable to feed fuel cell systems. H_2_ diffuses quicker in the anode than CO and the diffusion overvoltage is reduced [63].

The flow mixture between the biomethane and CeO_2_ ratio varies directly with syngas production. Increasing the biomethane content increases the CO flow rate and, thus, decreases the H_2_/CO ratio.

**Figure 7:**
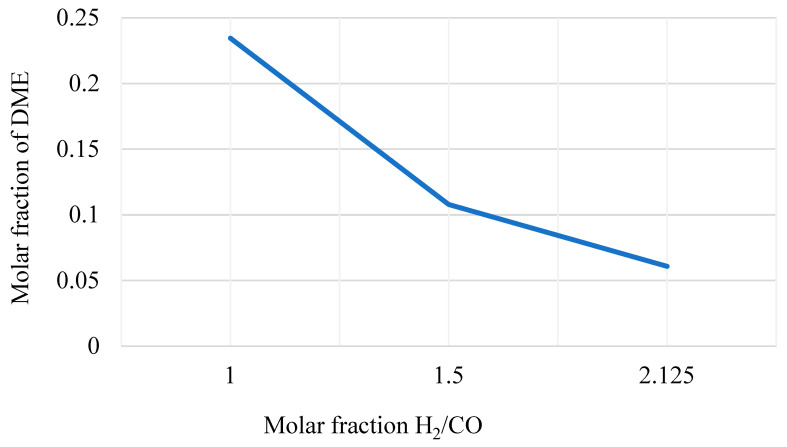
DME yield in the DME synthesis reactor varying the H_2_/CO molar ratio of the inlet streams.

The H_2_O/CO_2_ molar ratio is ≈1.13 for the oxidation reaction to obtain:

- The complete re-oxidation of Ce_2_O_3_ to CeO_2_;
- An H_2_/CO molar ratio around 1. This is an optimal value for the downstream DME production in the proper reactor (see Figure 7).

**Figure 8:**
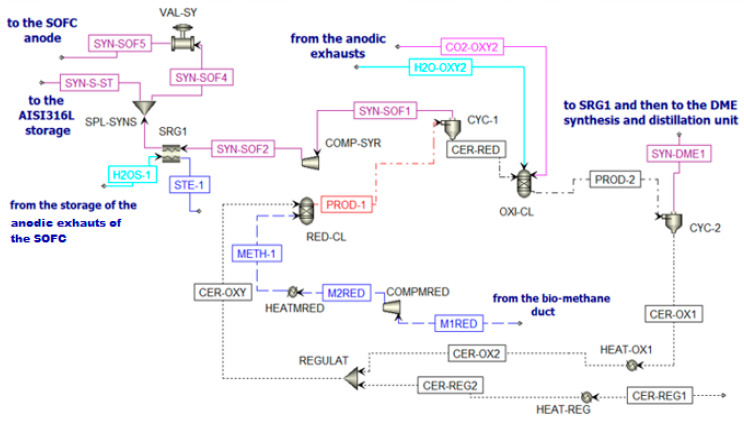
Chemical looping scheme in Aspen Plus.

Figure 8 reports the chemical looping implemented in Aspen Plus.

The inlet streams of the reduction reactor (RED-CL) are constituted by biomethane (METH-1) and CeO_2_ (CER-OXY). Particle size is defined from the particle distribution reported in Supplementary Table S1 [64]. At the outlet of this reactor, the flow PROD-1 is separated in cyclone CYC-1. Two different streams are produced. The first stream (Ce_2_O_3_ (CER-RED)) is sent to the oxidation reactor, while the second stream is the syngas fraction (SYN-SOF1). This stream is compressed and cooled to be stored in the AISI316L tank [65]. This fraction is stored at 10 bar and 800 °C, following the mechanical and physical properties of AISI316L. However, in Aspen Plus, there is no model for the storage system simulation. Thus, downstream COMP-SYR and SRG1, there is a splitter (SPL-SYNS), which separates the flow entering the anode of the SOFC (SYN-SOF4) from the flow that should remain in the storage (SYN-S-ST), and a valve (VAL-SY) that reduces the pressure of the portion of syngas sent to the SOFC at 5 bar (SYN-SOF5). To sum up, for a continuous operation of the SOFC, the SYN-SOF1 mole flow is spread over the whole year and the molar flow stream SYN-SOF4 is obtained according to Equation (5).

(5) $${\dot{n}}_{\mathrm{SYN}-\mathrm{SOF}4}\;=\;{\dot{n}}_{\mathrm{SYN}-\mathrm{SOF}1}\;\left(\frac{\mathrm{kmol}}{\min}\right)\;\frac{\mathrm{operating}\;\mathrm{miuntes}\;\mathrm{of}\;\mathrm{the}\;\mathrm{CL}}{\mathrm{operating}\;\mathrm{minutes}\;\mathrm{of}\;\mathrm{the}\;\mathrm{SOFC}}:\;60\;\frac{s}{\min}$$

Going back to the chemical looping unit, the oxidation reactor (OXY-CL) is fed by the reduced cerium oxide (CER-RED) and a mixture of H_2_O and CO_2_ coming from the anodic exhausts of the SOFC. The product of the oxidation reactor (PROD-2) goes to a cyclone CYC-2 to separate the solid fraction (CeO_2_) and the gaseous fraction (SYN-DME1). The solid flow is sent to the reduction reactor to close the cycle (CER-OX1) after a re-integration of the lost ceria (CER-REG2) for the nonunitary efficiency of the cyclones (Supplementary Table S2). The gaseous flow is sent to the DME production section of the plant.

#### 2.2.2. Solid Oxide Fuel Cell (Secondary Device)

**Figure 9:**
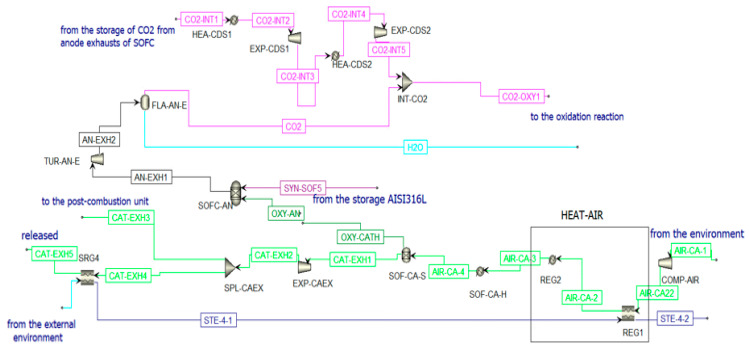
SOFC scheme in Aspen Plus when the CL is in ON state.

To produce electricity, a fuel cell is used to consider its higher efficiency and fuel flexibility compared to a thermal machine [66]. Particularly, an anode-supported planar Solid Oxide Fuel Cell is chosen for its advantages compared to the other fuel cells in the industry [67,68,69,70]. The SOFC is simulated in Aspen Plus for both situations when the CL is in the ON-state (Figure 9) and OFF-state (Supplementary Figure S1).

The operating conditions of the SOFC system are:

(1) Temperature = 850 °C, which is compatible with syngas storage;
(2) Pressure = 5 bar, to improve the cell’s performance [71].

The SOFC operating temperature could be lowered using new and advanced materials. It is selected as a condition suitable for the bloom energy generator [72].

As specified in Section 2.2.1, the syngas mole flow entering the anode of the SOFC is $\dot{n}$_SYN-SOF5_; another inlet stream in the anode is given by the oxygen ions (OXY-AN). The molar flow rate of this stream is equal to the stoichiometric molar flow rate of the oxygen O_2_ needed to generate in the cathode an amount of ions O^2−^ sufficient to oxidize the fuel. It is calculated according to the Faraday law (4):

(6) $${\dot{\;n}}_{\mathrm{oxy}-\mathrm{an}}=\frac{C_{tot}}{Z_{O_{2}}\;\to \;F}$$

where $C_{tot}$ = current produced by the cell and $Z_{O_{2}}$ = 4, so $\dot{\;n}$_oxy-an_ = 148.42 $\frac{\mathrm{mol}}{s}$. This molar flow rate of oxygen is set equal to the molar flow rate of the stream OXY-CATH exiting from the component SOF-CA-S to simulate the conduction of the ions from the anode to the cathode in the electrolytic layer of the SOFC. The cathode is supplied by air that enters the plant at ambient conditions to then be compressed and heated to 5 bar and 600 °C. The molar flow rate of the air stream is calculated iteratively to be heated from 600 °C to 850 °C, absorbing the entire heat rejected by the SOFC [73]: $\dot{\;n}$_AIR-CA-3_ = 3.044 $\frac{\mathrm{kmol}}{s}$. Once the air stream (AIR-CA-3) enters the cathode, it first encounters a heater that simulates the heating of the air and then a separator in which the streams OXY-CATH and CATH-EXH1 are produced. The latter represents the cathodic exhausts. They are expanded and then split into two streams. One stream (CAT-EXH4) feeds an HRSG (SRG4) to produce steam before the external release (CAT-EXH5); the other stream (CAT-EXH3) feeds the post-combustion unit. Considering a cell voltage equal to around 0.8 V, the Solid Oxide Fuel Cell can produce an electric power equal to 45.82 MW_e_. Using the Bloom Energy Servers [72] with an electric power production of 200 kW, the number of necessary modules is 230.

#### 2.2.3. DME Production (Secondary Device)

##### DME Synthesis

The single-step pathway with a dual catalyst and the co-production of methanol and DME is chosen for the DME synthesis. This process is characterized by a significant increase in the total methanol yield compared to the two-step pathway with methanol and DME produced in two different reactors [74]. Three main reactions occur: syngas conversion to methanol (reaction (7)), water gas shift (reaction (8)), and methanol dehydration to DME (reaction (9)).

CO_2_ + 3H_2_ → CH_3_OH + H_2_O    ΔH^0^ = −49.2 kJ/mol (7)

CO + H_2_O → CO_2_ + H_2_                ΔH^0^ = −41.2 kJ/mol (8)

2CH_3_OH → CH_3_OCH_3_ + H_2_O   ΔH^0^ = −24.0 kJ/mol (9)

The global reaction is:

3H_2_ + 3CO → CH_3_OCH_3_ + CO_2_    ΔH^0^ = −246.0 kJ/mol (10)

**Figure 10:**
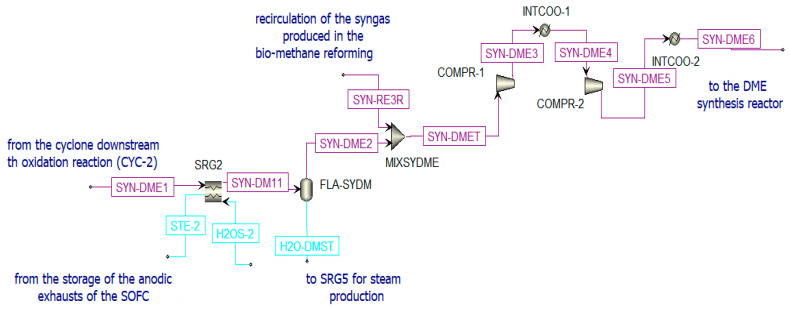
Pre-treatment of the syngas sent to the DME reactor in Aspen Plus.

According to the Le Châtelier principle [42], the global reaction is favored at high pressure and low temperature. The chosen catalytic reactor is a multi-tube fixed-bed reactor that is kept at a constant temperature of 250 °C and at 50 bar [75]. The bi-functional catalyst Cu/ZnO/Al_2_O_3_: γ-Al_2_O_3_ is selected considering a loading ratio of 1:2 from the literature. The syngas produced in the oxidation reactor of the chemical looping (SYN-DME2), before being sent to the DME synthesis reactor (see Figure 10), is:

- Cooled in the heat recovery steam generator (SRG2), producing steam;
- Separated from water (in FLA-SYDM);
- Integrated with additional syngas (SYN-RE3R) produced in the biomethane reforming reactor and compressed to the operating pressure.

**Figure 11:**
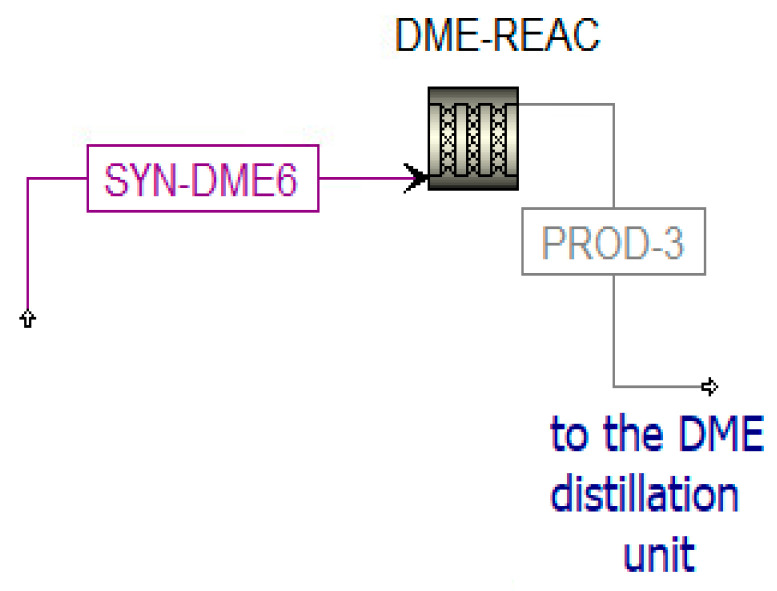
Simulation of the DME reactor in Aspen Plus.

The DME reactor is simulated in Aspen plus (Figure 11) and solved with the SRK-EOS property method. The parameters for the modeling of this component are listed in Table 2 [53].

A Langmuir–Hinshelwood–Hougen–Watson (LHHW) kinetic model is implemented considering the three simultaneous reactions defined above ((7), (8), and (9)). The expressions of the rates for CO_2_ hydrogenation, WGS, and methanol dehydration are calculated according to Equations (11)–(13), respectively [74,76]. These reaction rates are expressed in $\frac{\mathrm{kmol}}{{\mathrm{kg}}_{\mathrm{cat}}\cdot s}$:

(11) $$r_{CO_{2},hydrogenation}=\frac{k_{1}\;\left(p_{H_{2}}\cdot p_{CO_{2}}\right)\;\left[1-\left(\frac{1}{k_{eq,1}}\right)\cdot \frac{p_{CH_{3}OH}\cdot p_{H_{2}O}}{p_{CO_{2}}\cdot p_{H_{2}}^{3}}\;\right]}{\;{\left(1+k_{2}\cdot \frac{p_{H_{2}O}}{p_{H_{2}}}+\sqrt{k_{3}\cdot p_{H_{2}}}+k_{4}\cdot p_{H_{2}O}\right)}^{3}}$$

(12) $$r_{WGS}=\frac{k_{5}\cdot p_{CO_{2}}\;\left[1-\left(\frac{1}{k_{eq,2}}\right)\cdot \frac{p_{CO}\cdot p_{H_{2}O}}{p_{CO_{2}}\cdot p_{H_{2}}}\;\right]}{\;1+k_{2}\cdot \frac{p_{H_{2}O}}{p_{H_{2}}}+\sqrt{k_{3}\cdot p_{H_{2}}}+k_{4}\cdot p_{H_{2}O}}$$

(13) $$r_{MeOH,dehydration}=\frac{k_{6}\;K_{CH_{3}OH}^{2}\;\left[C_{CH_{3}OH}^{2}-C_{H_{2}O}\cdot \frac{C_{DME}}{K_{eq,3}\;}\;\right]}{\;{\left(1+2\;\sqrt{k_{CH_{3}OH}\cdot C_{CH_{3}OH}\;}+k_{H_{2}O}\cdot C_{H_{2}O}\right)}^{4}}$$

with:

- p, gas partial pressure (Pa);
- C, concentration (kmol/m^3^).

The parameters inserted in Equations (11)–(13) are taken from the literature [53].

##### Distillation Unit

**Figure 12:**
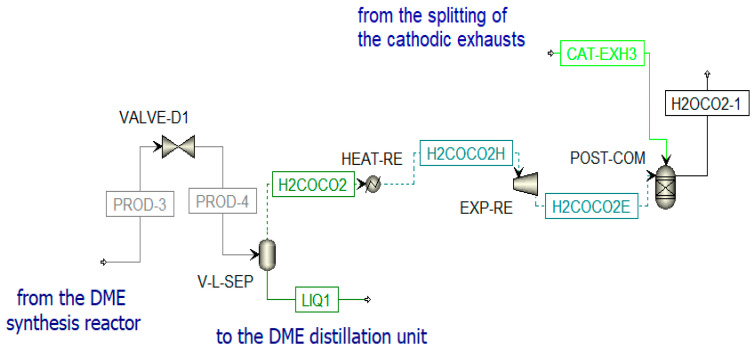
Post-combustion unit in Aspen Plus.

**Figure 13:**
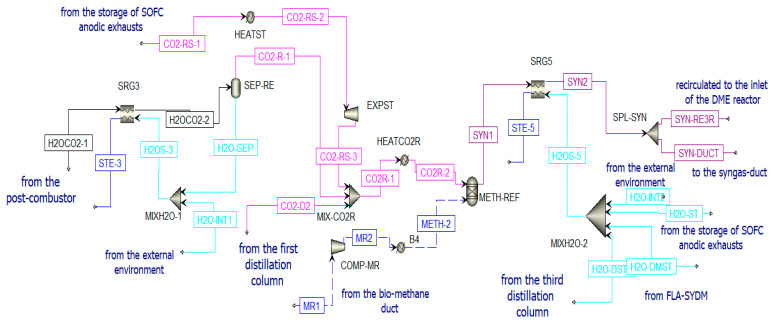
Treatment of H2OCO2-1 and reforming unit in Aspen Plus.

The DME synthesized in the reactor (PROD-3) is characterized by a high amount of impurities; thus, a separation process and a distillation process are necessary to obtain pure DME. Before the distillation columns, there is a vapor–liquid separation unit (V-L-SEP) at −45 °C and 10 bar, as shown in Figure 12. The output streams of this component are incondensable gases (H2COCO2) and a liquid stream (LIQ1). The first stream (H_2_COCO_2_) is pre-heated to 400 °C and expanded. The resulting stream is sent to a post-combustion chamber (POST-COM) (see Figure 12) to produce H2OCO2-1, which is cooled in a heat recovery steam generator (SRG3) to be separated in a flash unit (SEP-RE) (see Figure 13). The obtained water at the bottom of the reactor is used to produce steam in counter-flow with the post-combustion exhausts in the previously mentioned SRG3, while the gaseous stream feeds the biomethane reforming reactor (METH-REF) after being mixed with other streams of CO_2_. These additional streams come from the portion of the anodic exhausts of the SOFC (CO2-RS-3) stored when the chemical looping is in OFF-state and not used in the oxidation reactor of the CL itself throughout the year. Another stream that enters the reforming reactor is biomethane (METH-2), taken from the pipeline. The aim of the biomethane reforming reactor (METH-REF) is to produce syngas following this reaction:

(14) $${\mathrm{CH}}_{4}\;+\;{\mathrm{CO}}_{2}\to 2H_{2}\;+\;2\mathrm{CO}\;\;\Delta H^{0}\;=\;247.0\;\mathrm{KJ}/\mathrm{mol}$$

This reaction is favored at low pressures and high temperatures. In the present study, it takes place at 800 °C and 1 bar. It can be fed by concentrated solar thermal energy in an additional receiver-reactor placed close to the reduction reactor of the CL. The syngas stream obtained from this reactor (SYN1) is cooled in the heat recovery steam generator (SRG5) and split (in SPL-SYN) to be partially recirculated to the DME synthesis reactor (SYN-RE3R) and partially sent to a syngas duct (SYN-DUCT). This last stream is an additional output of the overall system.

**Figure 14:**
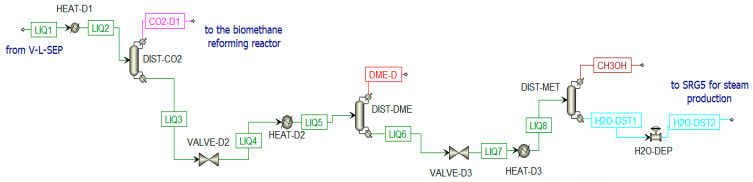
Distillation unit in Aspen Plus.

The second stream from V-L-SEP is a liquid stream mainly constituted of dissolved CO_2_, DME, and CH_3_OH (LIQ1). This stream is further treated in three different distillation columns (Figure 14):

1. Column for CO_2_ separation (DIST-CO2);
2. Column for DME production (DIST-DME);
3. Column for methanol separation from water (DIST-MET). The water stream (H2O-DST1) is mixed with other water streams for steam production in SRG5.

A valve and a heat exchanger are placed before each column to adjust the pressure to the optimal value and to have 50% of vapor in the column inlet stream [75]. The number of stages used in the distillation columns is estimated by increasing them until a certain change in composition is detected. In Table 3, the data inserted in the distillation columns are listed; while in Table 4 are listed the composition of thermodynamic properties of selected streams when the chemical looping is in ON-State.

##### Steam Production

In the analyzed plant, there are five heat recovery steam generators (SRG1, SRG2, SRG3, SRG4, and SRG5) with the primary purpose of cooling certain streams of the plant without wasting their high-temperature heat. In certain generators (SRG1 and SRG2), the water needs are completely satisfied through the recirculation of water produced in the system itself from different processes. In the others, the water is re-integrated from external sources. In Table 5, the steam streams produced in these HRSGs are listed.

##### Solar Tower and Heliostats

The reduction reactor of the chemical looping and the biomethane reforming reactor work simultaneously; they can be supplied by concentrated solar energy. As a result of the high temperatures and the overall thermal requirements (W_th,tot_ ~ 294 MW_t_), it is considered to use a solar tower system [77,78]. This system could be installed in Turin, with two receiver-reactors where the reduction and the oxidation take place. These receiver-reactors are similar to the receiver-reactor of the solar dish located on the Energy Center roof, both in terms of irradiance and temperature values. Furthermore, these data are available:

1. The experimental daily values of the direct normal irradiance in the neighborhood of the Energy Center, measured by the Politecnico meteorological station;
2. The experimental seasonal daily average temperatures of the receiver-reactor at the focus of the dish system installed on the Energy Center roof.

The yearly average direct normal irradiance (DNI) hitting the receiver-reactors and making the CL work properly is calculated to be around 828 $\frac{W}{m^{2}}$. Consequently, considering the optical efficiency of the solar field equal to 80%, its extension area should be around 44.27 hectares. To cover this area, 3170 heliostats with an aperture area of 140 m^2^ are necessary. The dimensions of the solar field are in the midpoint between those of the Khi Solar One system by Abengoa in the Northern Cape (South Africa) [79] and the Spanish Gemasolar system [80]. This aspect is highlighted because the electricity production of the polygeneration plant analyzed in this work is equal to the electricity production of the Spanish system (24.19 MW_e_ vs. 20 MW_e_), underlining the possible competitiveness of this plant with other systems supplied by concentrated solar energy.

### 2.3. Plant Operating Components in the Absence of a Sufficient Irradiance

When the chemical looping cannot operate properly (T_receiver-reactor_ < 900 °C), the plant operates as shown in Figure 4.

### 2.4. Thermal Balance of the Plant

The thermal balance is fundamental for assessing system costs. In the present study, the plant is analyzed at steady-state conditions. The thermal needs of the different units of the plant are evaluated.

#### 2.4.1. Thermal Analysis of the Chemical Looping

Solar energy satisfies the heat duty of the reduction reaction and the streams entering the reactor. On the other hand, the oxidation reaction is exothermic, and a fraction of heat can be used in other plant sections. In Supplementary Table S3, the heat duties of both these reactors are shown. The heating of the inlet flows of the oxidation reaction is executed exploiting a portion of the steam produced in SRG2 and SRG1 (Supplementary Figures S2–S4).

#### 2.4.2. Thermal Analysis of the SOFC

In the SOFC scheme, a large amount of heat is required to bring air to 600 °C. This thermal requirement (in HEAT-AIR) can be satisfied as follows:

1. Air is heated in REG1 from 200 °C to 265 °C through the steam produced in SRG4;
2. After this pre-heating, the air stream can be further heated to 600 °C in two different ways:
  (a) If the CL unit operates, air can be heated in REG2;
  (b) If the CL does not operate, air can be heated through an electric heater. This drastically lowers the plant efficiency.

#### 2.4.3. Thermal Analysis of the Reforming Unit

Different thermal flows are required for the biomethane reforming:

1. The vapor–liquid separation in V-L-SEP requires heat, and a steam portion produced in SRG5 is exploited (see Supplementary Figure S5);
2. The stream of CO_2_ coming from the storage of the anodic exhausts of the SOFC (CO2-RS-1) is heated in HEAT-ST through a steam portion produced in SRG5 (see Supplementary Figure S6);
3. Concentrated solar irradiance supplies the reforming reaction and heats the entering streams in the reactor. The thermal requirements of this section are listed in Supplementary Table S4.

#### 2.4.4. Thermal Analysis of the Distillation Unit

Before the entry into the different distillation columns, the liquid is heated through a portion of steam produced in SRG2 (see Supplementary Figures S7 and S8). Additionally, the reboilers of the distillation columns [81] are supplied by the thermal load produced in the post-combustion reactor.

## 3. Discussion of the Plant Results

### 3.1. Plant Efficiencies

In this section, different plant efficiencies are evaluated considering the two operating conditions.

#### 3.1.1. Electric Efficiency of the System

The net electric power produced from the plant is: W_EL,NET,CL-ON_ = 6.17 MW_e_ when the CL is in the ON-state and W_EL,NET,CL-OFF_ = 28.96 MW_e_ when the CL is in the OFF-state. The electric efficiency of the system is evaluated considering, respectively, the operation (15) or not (16) of the chemical looping:

(15) $$\eta_{electric,\;CL\;ON\;}=\frac{\;W_{\mathrm{EL},\mathrm{NET},\mathrm{CL}-\mathrm{ON}}\;\;}{\dot{Q_{RED-REACTOR}}+\dot{\;m_{METH-1}}\;\cdot \;LHV_{METHANE}+\dot{\;m_{METH-2}}\;\cdot \;LHV_{METHANE}+\dot{Q_{REFORMING}}}\;=0.6\%$$

(16) $$\;\eta_{electric,\;CL\;OFF\;}=\frac{\;W_{\mathrm{EL},\mathrm{NET},\mathrm{CL}-\mathrm{ON}}}{m_{SY\dot{N}-\mathrm{SOFC}}\cdot {\mathrm{LHV}}_{\mathrm{SYN}-\mathrm{SOFC}}}\;=26.41\%$$

The large difference between these two electric efficiencies is mainly related to the following relation $W_{EL,TOT,CL-OFF}$ >> $W_{\mathrm{EL},\mathrm{TOT},\mathrm{CL}-\mathrm{ON}}$. When the CL is in the OFF-state, steam streams can be exploited, and the following components are in the OFF-state (COMP-SYR, COMPR-1, and COMPR-2).

#### 3.1.2. Thermal Efficiency of the System

The waste steam and hot water streams produced in the five HRSGs could be mixed (see Supplementary Figures S9 and S10) and cooled to their initial temperature (15 °C) to produce thermal power. When the chemical looping is in the ON-state, W_th,tot,CL-ON_ = 111.97 MW_t_; otherwise, W_th,tot,CL-OFF_ = 35.82 MW_t_. The thermal efficiencies are, respectively, calculated as (17) and (18):

(17) $$\eta_{thermal,\;CL-ON\;}=\frac{W_{th,tot,CL-ON}\;}{\dot{Q_{RED-REACTOR}}+\dot{\;m_{METH-1}}\;\cdot \;LHV_{METHANE}+\dot{\;m_{METH-2}}\;\cdot \;LHV_{METHANE}+\dot{Q_{REFORMING}}\;}=\;10.94\%$$

(18) $$\eta_{thermal,\;CL-OFF\;}=\frac{W_{th,tot,CL-OFF}}{\dot{\;m_{SYN-SOFC}}\;\cdot \;LHV_{SYN-SOFC}}=\;32.67\%$$

This large difference is mainly related to the different fuels required by the system whether the CL is in the ON-state or OFF-state.

#### 3.1.3. Solar/Biomethane-to-Fuel Efficiency of the System

The fuel production only occurs when the CL operates.

(19) $$\eta_{SOLAR/BIOMETHANE-TO-FUEL,\;CL\;ON\;}=\frac{\;\;\dot{\;m_{DME}}\;\cdot \;LHV_{DME}+\dot{\;m_{METHANOL}}\;\cdot \;LHV_{METHANOL}+\dot{\;m_{SYN-DUCT}}\;\cdot \;LHV_{SYN-DUCT}\;\;}{\dot{Q_{RED-REACTOR}}+\dot{\;m_{METH-1}}\;\cdot \;LHV_{METHANE}+\dot{\;m_{METH-2}}\;\cdot \;LHV_{METHANE}+\dot{Q_{REFORMING}}\;}=\;51\%$$

(20) $$\eta_{SOLAR/BIOMETHANE-TO-FUEL,\;CL\;OFF\;}=\frac{\;0}{\dot{\;m_{SYN-SOFC}}\;\cdot \;LHV_{SYN-SOFC}}\;=\;0$$

#### 3.1.4. Global Efficiencies of the System

The global efficiencies of the plant in its two different operating conditions are evaluated:

(21) $$\eta_{global,\;CL-ON\;}=\eta_{electric,\;CL\;ON\;}\;\times \;\eta_{thermal,\;CL-ON\;}\;\times \;\eta_{SOLAR/BIOMETHANE-TO-FUEL,\;CL\;ON\;}=\;62.56\%$$

(22) $$\eta_{global,\;CL-OFF\;}=\eta_{electric,\;CL\;OFF\;}\;\times \;\eta_{thermal,\;CL-OFF\;}\;\times \;\eta_{SOLAR/BIOMETHANE-TO-FUEL,\;CL\;OFF\;}=\;59.08\%$$

These results highlight the slight decrease in the global efficiency of the polygeneration plant at low solar irradiances.

### 3.2. Results Discussion and Comparison with Similar Plant

The results obtained from the polygenerative plant are examined and compared with a similar study, by Farooqui et al. [53]. The main differences are:

1. The unit chosen for electric power production by Farooqui et al. [53] is a gas turbine. The thermal energy generated by the oxyfuel combustion chamber is used to operate continuously the chemical looping cycle; therefore, there is no exploitation of solar energy;
2. The chemical looping executed by Farooqui et al. [53] is not isothermal, the two reactions take place at different temperatures (1312 °C and 900 °C), and it occurs at a higher operating pressure (2 bar).

A comparison between the average yearly outputs and inputs of the two plants is shown in Table 6.

As can be seen in Table 6, the main difference between the present study plant remains in the lower electric power and DME production. This is strictly linked to the solar intermittence, which makes the chemical looping not continuous throughout the year. Consequently, although the size of the two plants is comparable, the chemical looping of this work produces a lower average yearly amount of syngas to be used for electricity and DME production. However, it is important to emphasize the high amount of thermal power produced, methanol and syngas, the lower amount of bio-methane needed, and the higher average global efficiency. In addition, among its outputs, there is no carbon dioxide to be sequestrated, because the plant completely reuses the produced CO_2_ and executes the CCU.

### 3.3. Improvement in the Productivity of the Plant

In the attempt to increase the plant productivity, it is thought to decouple the chemical looping operation from the intermittence of solar energy. The reduction reactor of the chemical looping is supplied with the thermal load deriving from the SOFC system. It is necessary to lower the reduction reaction temperature at 800 °C (the SOFC operates at 850 °C) and, consequently, for the complete ceria reduction, it is necessary to increase the amount of biomethane, sent to the reduction reactor (as seen in Figure 6), to 0.95. In this plant, the syngas storage system is eliminated because the SOFC anode can be continuously supplied by the reduction reactor of the CL (continuous working). Thus, the SOFC stacks can produce W_EL, SOFC_ = 243.48 MW_e_.

To fully eliminate the dependence of the polygeneration plant on the solar energy source, it is necessary to introduce a biomethane oxyfuel combustion unit.

The total biomethane requirement of this new unit is: $\dot{m}$_BIO-METH-THERMAL-TOT_ = 5.57 kg/s. This stream is sent to the oxyfuel combustion, according to reaction (23).

0.95CH_4_ + 0.05CO_2_ + 1.9O_2_ → CO_2_ + 1.9H_2_O (23)

Thus, the total amount of O_2_ required to burn $\dot{m}$_BIO-METH-TOT_ is $\dot{m}$_O2-required_ = 19.48 kg/s. Biomethane oxyfuel combustion, as compared to direct fuel combustion [82,83], leads to combustion exhausts mainly constituted of H_2_O and CO_2_. However, to obtain pure O_2_, an Air Separation Unit is necessary. The specific energy expenditure of the Air Separation Unit, to produce an O_2_ stream with 98% purity, can be assumed equal to 925 kJ/kg_O2_ [84,85]. As a result, the total power expenditure of the ASU is 18.02 MW_e_. The new plant performances on a yearly basis are shown in the first column of Table 7. Comparing the new plant with the solar-aided plant and the plant of Farooqui et al. [53], the new system is the best from the point of view of electricity, DME, methanol, and syngas production. However, there is a much higher amount of biomethane consumption. Additionally, both Farooqui et al.’s plant [53] and the newly studied plant have the non-negligible issue of CO_2_ sequestration. The biomethane requirement of the new plant could be reduced by eliminating the reforming unit, even if the CO_2_ produced by the plant increases. This occurs because, beyond the CO_2_ production from the biomethane oxyfuel combustion, the distillation and the SOFC units contribute to CO_2_ production. The syngas lacks and CO_2_ production decreases the plant efficiency (even if the produced electric power is the highest). The performance of this plant is shown in the second column of Table 7.

From a pure planning point of view, there is no absolute optimum solution for the choice of the plant; this choice depends on the main goal:

1. If there is a need for a medium-sized plant (W_el,average requirement_ < 50 MW) to be installed in a location with a high availability of solar energy and space, the best choice would be the first polygeneration system analyzed with CS integration and no CO_2_ emissions;
2. If a large-sized plant is required (W_el,average requirement_ > 100 MW), with a high availability of biomethane, the best system is the new plant analyzed with the integration of the reforming unit, proving that adequate sequestration of the CO_2_ produced is achieved;
3. If a large-sized plant is required (W_el,average requirement_ > 100 MW), but no biomethane is available to feed the system, the optimal solution is the new system analyzed without the integration of the reforming unit. However, in the latter case, it is important to have an adequate and much larger storage system for the CO_2_ sequestering.

Considering the fuel production, the preferred system is the new system without solar energy integration but with the reforming unit; the other two systems are comparable.

## 4. Evaluation of the Chemical Looping Model Performance

This section focuses on the evaluation of the chemical looping performance comparing the results obtained from the CL model of the present study with other experimental [54] and modeling [41,55] literature studies. The present study simulates the CeO_2_/Ce_2_O_3_ chemical looping using two Gibbs reactors [41]. Thus, only the thermodynamics of the reactions is considered, and the kinetic limitations of the chemical reactions are neglected. The discrepancy between the thermodynamic simulation and the actual chemical reactions can be seen by comparing the model results of this work with the experimental study of the combined reduction of ceria and methane reforming in a particle transport reactor driven by solar energy [54]. However, these experiments [54] are executed at a temperature equal to 1302 °C (Table 8).

The main points of difference between thermodynamic simulation and real experiments are:

1. Absence of carbon deposits in the model. Thermodynamically, the phenomenon is verifiable at methane-to-ceria ratios above 1 and a temperature above 900 °C [41];
2. High methane conversion compared to the ideal case.

It follows that the evaluation of the chemical looping performance is made by considering other similar thermodynamic models, such as Bose et al. [41] and Warren et al. [55]. An agreement between the results is obtained, as shown in Supplementary Tables S5–S7. The operating conditions between Bose and the present study are similar, with an operating temperature of 900 °C and a ratio of 0.8 (CH_4_/CeO_2_). The molar concentration of carbon monoxide is similar, while the hydrogen fraction is slightly higher for Bose. This is due to a more uniform temperature distribution.

## 5. Conclusions

This study aims to evaluate the performance of a polygeneration plant to produce heat, electricity, DME, syngas, and methanol with the integration of a chemical looping CeO_2_/Ce_2_O_3_ driven by solar energy and coupled to the biomethane reforming system. Some advantages of this system can be listed below:

1. The plant using only renewable energy sources contributes to the industrial decarbonization;
2. A carbon capture and utilization unit is implemented. The system is very versatile and environmentally sustainable while still relying on fossil fuel energy production to a small extent. The SOFC unit could be replaced by an existing fossil-fuel power plant, whose effluent can be sent to the CL oxidation reactor and the reforming unit;
3. Production of innovative, green fuels that can help reduce the dependence on liquefied petroleum gases;
4. The use of the SOFC stack for electricity and heat generation. The efficiency of these systems is high, especially considering the electric share;
5. The production of syngas as an energy carrier to store and exploit the solar aleatoriness.

However, the coupling between the chemical looping and a solar energy system produces intermittence in the CL system. The absence of solar exploitation leads to a more complex system with greater production of electricity and green fuels. The following consequences arise:

1. Higher biomethane consumption;
2. CO_2_ production in the oxyfuel unit. This leads to problems with the CO_2_ produced.

Considering the decarbonization, the energy transition, and the reduction of greenhouse emissions as the main focus, the best system to install would be a polygeneration plant with the integration of a solar-powered chemical looping. Further studies could be conducted on this system, such as:

  - Evaluation of a kinetic model for chemical looping operation;
  - An economic analysis to assess the net present value and payback time of the investment;
  - An exergetic analysis to identify the components that can be improved in the complex process;
  - A more detailed assessment of the environmental impact and a discussion related to the material recovery of the various components.

## Figures and Tables

**Table 1:** Main assumptions and hypotheses adopted in the process simulation.

**Biomethane**			95% CH_4_, 5% CO_2_		
**Oxidation and reduction reactors**			Model: RGIBBS, no heat losses		
**Compressors, pumps, and turbines**			$\eta_{is,comp}=0.9\;\eta_{mech,comp}=$ 0.98 $\eta_{is,\;tur}=0.9\;\eta_{mech,tur}$= 0.98 $\eta_{is,pump}\;$= 0.9 $\eta_{driver,pump}\;$= 0.9		
**SOFC**			Separator and heat exchanger for the SOFC cathode Model: RGibbs for the SOFC anode		
**Methane reforming**			Model: RGibbs, no heat losses		
**Oxygen carrier**			CeO_2_, Ce_2_O_3_, a temperature drop of 20 °C from OXY-CL to RED-CL		
**DME reactor**			Operation T = 250 °C, *p* = 50 bar, Model: RPLUG multi-tube reactor		
**Distillation unit**			Reboiler type: Kettle, Model: RADFRAC		
**DIST-CO_2_**		**DIST-DME**		**DIST-MET**	
*p* = 10 bar		*p* = 9 bar		*p* = 2 bar	

**Table 2:** Parameters of the DME reactor.

N° Tubes	Diameter (m)	Bed Voidage	Density Cu/ZnO/Al_2_O_3_ (kg/m^3^)	Density γ-Al_2_O_3_ (kg/m^3^)	ρ_average_ (kg/m^3^)	Temperature (°C)	Pressure (bar)	Length (m)
5500	0.02	0.45	1200	1470	1380	250	50	15

**Table 3:** Distillation columns’ operation parameters.

	Pressure (bar)	T_REB_ (°C)	Q_REB_ (MW)	T_COND_ (°C)	Q_COND_ (MW)	Number of Stages	Feed-in Stage	Purity of the Product
DIST-CO2	10	49.64	1.1	−40.58	−1.5	25	10	-
DIST-DME	9	140.73	2.7	45.18	−1.7	30	24	98%
DIST-MET	2	113.63	1	82.89	−0.8	24	18	99%

**Table 4:** Composition and thermodynamic properties of selected streams when the CL is in ON-STATE. The output material streams of the plant are highlighted.

Stream	P (bar)	T (°C)	Mole Flow (kmol/s)	Molar Fraction									
				Ce_2_O_3_	CeO_2_	CH_4_	H_2_O	CO_2_	CO	H_2_	N_2_	CH_3_OH	DME
CER-OXY	1	900	0.59	0	1	0	0	0	0	0	0	0	0
CER-RED	1.19	900	0.27	1	0	0	0	0	0	0	0	0	0
SYN-SOF1	1.19	900	1.14	0.03	0	0.11	0	0	0.30	0.56	0	0	0
CO2-OXY2	1	900	0.19	0	0	0	0.01	0.79	0.07	0.13	0	0	0
H2O-OXY2	1	900	0.18	0	0.07	0	0.93	0	0	0	0	0	0
SYN-DME1	1.18	900	0.42	0	0.13	0	0.09	0.06	0.34	0.38	0	0	0
SYN-DME6	50	250	1.18	0	0	0	0.04	0.06	0.46	0.43	0.01	0	0
PROD-3	50	250	0.58	0	0	0.01	0.01	0.42	0.11	0.16	0.02	0.04	0.23
CO2R-2	1	500	0.71	0	0	0.06	0.01	0.77	0.05	0.07	0.04	0	0
METH-2	1	700	0.42	0	0	0.95	0	0.05	0	0	0	0	0
**SYN-DUCT**	**1**	**30**	**1.15**	**0**	**0**	**0.01**	**0.03**	**0.04**	**0.48**	**0.44**	**0**	**0**	**0**
**DME-D**	**9**	**45**	**0.13**	**0**	**0**	**0**	**0**	**0.01**	**0**	**0**	**0**	**0.01**	**0.98**
**CH3OH**	**2**	**83**	**0.02**	**0**	**0**	**0**	**0**	**0**	**0**	**0**	**0**	**1**	**0**

**Table 5:** Cold sides of the HRSGs.

HRSG	Water Requirements		
	$\mathrm{Mole}\;\mathrm{Flows}\;\left(\frac{kmol}{min}\right)$	Outlet Temperature (°C)	$\mathrm{Additional}\;\mathrm{Water}\;\mathrm{from}\;\mathrm{the}\;\mathrm{External}\;\mathrm{Environment}\;\left(\frac{\mathrm{kmol}}{\min}\right)$
SRG1	22.16	1326.5	0
SRG2	13.58	546.5	0
SRG3	4.32	381.4	3.5
SRG4	41.55	475.2 when CL does not work	41.5
		463.9 when CL works	
SRG5	40.40	748.2	35
**Total additional water requirement** $\left(\frac{\mathrm{kmol}}{\min}\right)$			80.1

**Table 6:** Comparison between the yearly average outputs of the polygeneration plant of this paper and Farooqui et al.’s study [53].

	Farooqui et al. [53]	Present Study
Biomethane feed (kton/year)	220.7	102.3
W_el,net,average_ (MW_e_)	102.9	24.2
W_th,average_ (MW_t_)	0	51.8
$\dot{m}$_DME_ (kton/year)	67.8	40.8
$\dot{m}$_MeOH_ (ton/year)	946.1	4686.4
Captured CO_2_ to be sequestrated (kton/year)	271.8	0
Syngas (kton/year)	0	129.9
$\eta_{tot,ave}$ (%)	50.2	59.8

**Table 7:** Performances of the new non-solar plants on yearly basis.

	Farooqui et al. [53]	New Plant without CS	New Plant without CS and Reforming
Biomethane feed (kton/year)	220.75	729.5	425.2
$W_{\mathrm{solar}\;\mathrm{field},\mathrm{ave}}$ (MW_t_)		0	0
W_el,net,average_ (Mwe)	102.9	119.7	144.4
W_th,average_ (MWt)	0	0	0
$\dot{m}$_DME_ (kton/year)	67.8	189.3	82.7
_MeOH_ (ton/year)	946.08	41510.8	9703.6
Captured CO_2_ to be sequestrated (kton/year)	271.84	444.7	797.6
Syngas (kton/year)	0	739.0	0
$\eta_{tot,ave}$ (%)	50.21	66.1	35.6

**Table 8:** Comparison between the results of the reduction reactor model of this paper and the experimental data in the literature [54].

Reduction Reaction Set-Up		
**Temperature (°C)**	1302	
$\dot{n_{CeO_{2}}}$. $\left(\frac{\mathrm{mmol}}{\min}\right)$	44.2	
$\dot{n_{CH_{4},0}}$ $\left(\frac{\mathrm{mmol}}{\min}\right)$	9	
**Products composition at the steady-state**		
**Component**	**Present study**	**Welte, Warren, and Scheffe** [54]
$\dot{n_{H_{2}}}$ $\left(\frac{\mathrm{mmol}}{\min}\right)$	10.85	14
$\dot{n_{CO}}$ $\left(\frac{\mathrm{mmol}}{\min}\right)$	7.49	6
$\dot{n_{CO_{2}}}$ $\left(\frac{\mathrm{mmol}}{\min}\right)$	1.51	0.24
$\dot{n_{H_{2}O}}$ $\left(\frac{\mathrm{mmol}}{\min}\right)$	6.25	0.75
$\dot{n_{CH_{4}}}$ $\left(\frac{\mathrm{mmol}}{\min}\right)$	0	1.4
$\dot{n_{C}}$ $\left(\frac{\mathrm{mmol}}{\min}\right)$	0	1.2
**Methane conversion**		
$x_{CH_{4}}$ = 1 − $\frac{\dot{n_{CH_{4}}}}{\dot{n_{CH_{4},0}}}$	1	0.85

## Data Availability

Original data can be requested to the corresponding author.

## Supplementary Materials

The following supporting information can be downloaded at: https://www.mdpi.com/article/10.3390/ma16010315/s1, Figure S1: SOFC scheme in Aspen Plus when the CL is in OFF-state; Figure S2: Thermal balance of HEA-CDS1 and HEA-CDS2 in Aspen Plus; Figure S3: Thermal balance of HEAT-CO2 in Aspen Plus; Figure S4: Thermal balance of HEAT-H2O in Aspen Plus; Figure S5: Thermal balance of HEAT-RE in Aspen Plus; Figure S6: Thermal balance of HEAT-ST in Aspen Plus; Figure S7: Thermal balance HEAT-D2 in Aspen Plus; Figure S8: Thermal balance of HEAT-D3 in Aspen Plus; Figure S9: Mixer of the steam and hot water streams of the plant and simulation of the cooling of the output stream (STE-TOT) to obtain useful thermal power (THE-REQ) when CL is in ON-state; Figure S10: Simulation of the thermal recovery from steam when CL is in OFF-state; Table S1: Particle size distribution of the ceria as a function of the frequency obtained from ELPI measurements [63]; Table S2: Cyclones’ characteristics in Aspen Plus [65]; Table S3: Thermal balance of the chemical looping; Table S4: Thermal balance of the reforming unit; Table S5: Comparison of results of the reduction reactor model of this paper with the results reported by Bose et al. [36] at the chosen operating conditions of the solar aided CL; Table S6: Comparison of the results of the reduction reactor model of this study with the results reported by Warren et al. [54] and by Bose et al. [36] at different operating conditions; Table S7: Comparison of the results of the model of this study with the results reported by Bose et al. [36] for the oxidation of ceria with H_2_O and CO_2_.

## Nomenclature

CCU	Carbon Capture and Utilization
CL	Chemical Looping
CS	Concentrated Solar energy
CSP	Concentrated Solar Panels
DME	Dimethyl-ether
DNI	Direct Normal Irradiance
HRSG	Heat Recovery Steam Generator
SOFC	Solid Oxide Fuel Cell
LPG	Liquefied Petroleum Gases
TSCs	Thermochemical Splitting Cycles

## Units of measure

$\dot{m}$	mass flow rate, kg/s
$\dot{n}$	molar flow rate, mol/s
ΔT	phase change temperature range, °C
l	length, m
p	pressure, bar
r	reaction rate, kmol/(kg_cat_·s)
*T*	temperature, °C
W_el_	electric power, W_e_
W_th_	thermal power, W_t_
ρ	density, kg/m^3^
