# Supplementary material for: Modeling Analysis of a Polygeneration Plant Using a CeO2/Ce2O3 Chemical Looping
Source: Materials (Basel). 2022 Dec 29;16(1):315. doi: 10.3390/ma16010315 (PMC9822425; doi:10.3390/ma16010315)
Supplement: Supplementary file 1 [file materials-16-00315-s001.zip › materials-1909677-supplementary.pdf]

materials

MDPI

Supplementary Material

Modeling Analysis of a Polygeneration Plant Using a CeO_2/Ce_2O_3 Chemical Looping

Greta Magnolia [1], Massimo Santarelli [1], Domenico Ferrero [1] and Davide Papurello [1,2,*]

[1] Department of Energy (DENERG), Politecnico di Torino, Corso Duca Degli Abruzzi, 24, 10129 Turin, Italy

[2] Energy Center, Politecnico di Torino, Via Borsellino 38/18, 10129 Turin, Italy

* Correspondence: davide.papurello@polito.it; Tel.: +39-3402351692

Table S1. Particle size distribution of the ceria as a function of the frequency obtained from ELPI measurements [63].

Particle diameter	Frequency of particles (%)
95 nm–264 nm	11.4
264 nm–384 nm	17
384 nm–616 nm	35
616 nm–953 nm	23
953 nm–1.61 μm	9
1.61 μm–2.4 μm	3
2.4 μm–4.01 μm	1
4.01 μm–6.71 μm	0.23
6.71 μm–9.96 μm	0.37

Table S2. Cyclones' characteristics in Aspen Plus [65].

Calculation method	Leith-Licht
**Type**	Stairmand-HE
Separation efficiency	0.9
Maximum pressure drop	0.015 bar
Maximum number of cyclones	100

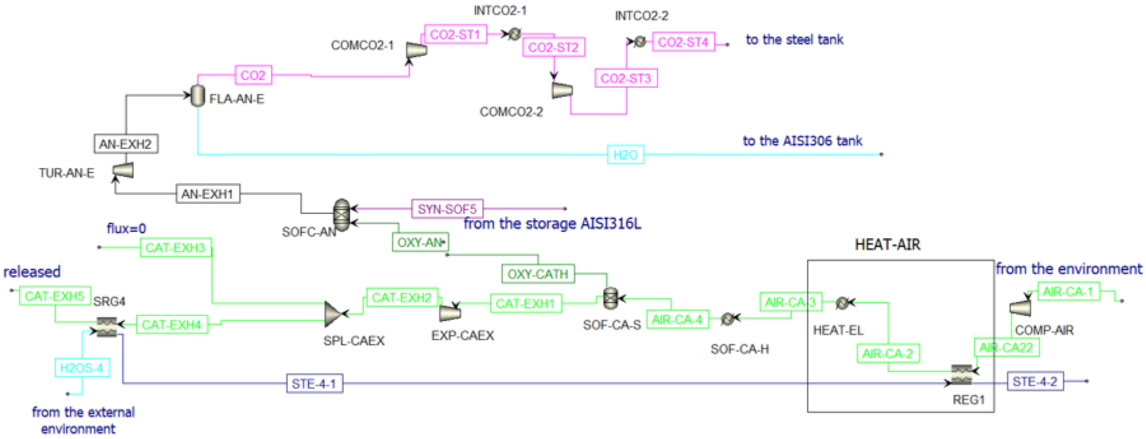

Figure S1. SOFC scheme in Aspen Plus when the CL is in OFF-state.

Table S3. Thermal balance of the chemical looping.

COMPONENT	HEAT DUTY [MW_t]	
**Reduction reactor**	Reduction reaction	−114.0
	Methane stream	−20.12
	Total ceria stream	−7.39
**Oxidation reactor**	32.97	

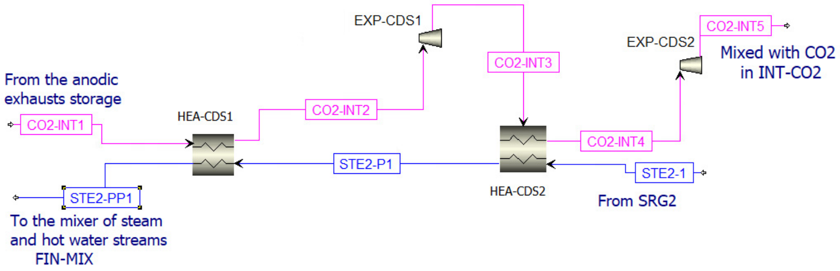

Figure S2. Thermal balance of HEA-CDS1 and HEA-CDS2 in Aspen Plus.

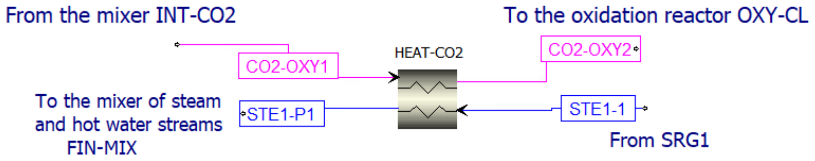

Figure S3. Thermal balance of HEAT-CO2 in Aspen Plus.

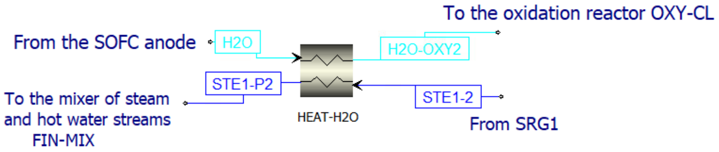

Figure S4. Thermal balance of HEAT-H2O in Aspen Plus.

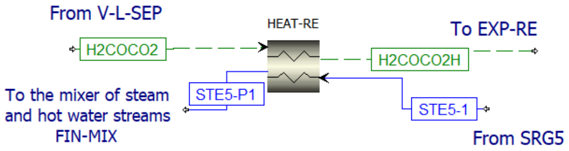

Figure S5. Thermal balance of HEAT-RE in Aspen Plus.

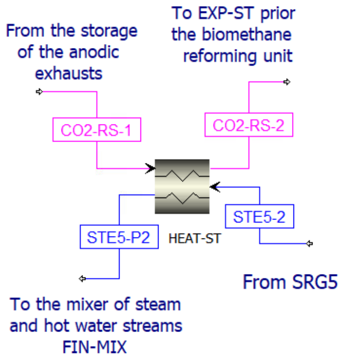

Figure S6. Thermal balance of HEAT-ST in Aspen Plus.

Table S4. Thermal balance of the reforming unit.

COMPONENT	HEAT DUTY [MWₜ]	
**Reforming reactor**	Reforming reaction	−126.74
	Methane stream	−11.53
	CO_2 stream	−14.15

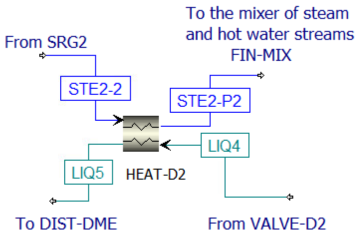

Figure S7. Thermal balance HEAT-D2 in Aspen Plus.

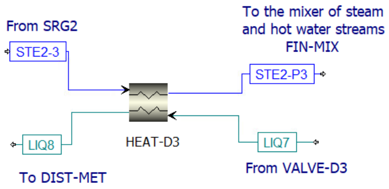

Figure S8. Thermal balance of HEAT-D3 in Aspen Plus.

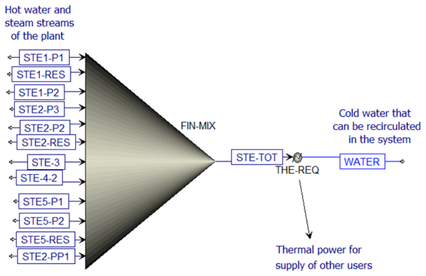

Figure S9. Mixer of the steam and hot water streams of the plant and simulation of the cooling of the output stream (STE-TOT) to obtain useful thermal power (THE-REQ) when CL is in ON-state.

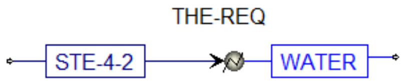

Figure S10. Simulation of the thermal recovery from steam when CL is in OFF-state.

Table S5. Comparison of results of the reduction reactor model of this paper with the results reported by Bose et al. [36] at the chosen operating conditions of the solar aided CL.

**Reduction reaction, p = 1 bar**		**Bose et al.** [36]	**Present study**
Operating conditions	Temperature	900–950 °C	900 °C
	CH_4/CeO_2	0.7–0.8	0.8
Produced syngas	H_2	63%	55%
	CO	31%	31%

Table S6. Comparison of the results of the reduction reactor model of this study with the results reported by Warren et al. [54] and by Bose et al. [36] at different operating conditions.

**Reduction reaction, CH_4/CeO_2 = 0.25 p = 1 bar**					
**Temperature (°C)**	**Mole fraction of exit gas**				
	**H_2**	**CO**	**CH_4**	**H_2O**	**CO_2**
**900 °C**					
**Warren et al.** [54]	0.718	0.273	0.004	0.004	0
**Bose et al.** [36]	0.655	0.329	0.006	0.009	0.001
**Present study**	0.585	0.400	0.007	0.009	0.004
**1000 °C**					
**Warren et al.** [54]	0.699	0.301	0	0	0
**Bose et al.** [36]	0.639	0.325	0	0.028	0.008
**Present study**	0.572	0.400	0	0.028	0.010

Table S7. Comparison of the results of the model of this study with the results reported by Bose et al. [36] for the oxidation of ceria with H2O and CO2.

**Oxidation reaction, $\dot{n}_{CeO_2} = 0.5\ \frac{kmol}{h}$, p = 1 bar**					
**Temperature (°C)**	**Waste gas flow $\left(\frac{kmol}{h}\right)$**	**Mole fraction of exit gas**			
			**H_2**	**CO**	**Other gases + CeO_2 residuals**
900 °C	0.5	Bose et al. [36]	0.440	0.450	0.110
		Present study	0.406	0.411	0.183
	0.75	Bose et al. [36]	0.280	0.320	0.400
		Present study	0.280	0.303	0.417
	1	Bose et al. [36]	0.210	0.240	0.550
		Present study	0.212	0.238	0.550

References

63 Andersson, M.; Yuan, J.; Sundén, B. SOFC modeling considering electrochemical reactions at the active three phase boundaries. *Int. J. Heat Mass Transf.* **2012**, *55*, 773–788. https://doi.org/10.1016/j.ijheatmasstransfer.2011.10.032.

65 Stainless Steel Type 316/316L, Rolled Metal Products | Stainless, Aluminum & Specialty Alloys. Available online: https://rolledmetalproducts.com/stainless-steel-type-316316l/ (accessed on 21 November 2022).

36 Xiao, l.; Wu, S.-Y.; Li, Y.-R. Advances in solar hydrogen production via two-step water-splitting thermochemical cycles based on metal redox reactions. *Renew. Energy* **2012**, *41*, 1–12. https://doi.org/10.1016/j.renene.2011.11.023.

54 Welte, M.; Warren, K.; Scheffe, J.R.; Steinfeld, A. Combined Ceria Reduction and Methane Reforming in a Solar-Driven Particle-Transport Reactor. *Ind. Eng. Chem. Res.* **2017**, *56*, 10300–10308. https://doi.org/10.1021/acs.iecr.7b02738.
